# Hepatitis B Virus DNA Integration, Chronic Infections and Hepatocellular Carcinoma

**DOI:** 10.3390/microorganisms9081787

**Published:** 2021-08-23

**Authors:** Maria Bousali, George Papatheodoridis, Dimitrios Paraskevis, Timokratis Karamitros

**Affiliations:** 1 Bioinformatics and Applied Genomics Unit, Department of Microbiology, Hellenic Pasteur Institute, 11521 Athens, Greece; mbousali@gmail.com; 2 Department of Gastroenterology, “Laiko” General Hospital of Athens, Medical School, National and Kapodistrian University of Athens, 11527 Athens, Greece; gepapath@med.uoa.gr; 3 Department of Hygiene Epidemiology and Medical Statistics, School of Medicine, National and Kapodistrian University of Athens, 15772 Athens, Greece; dparask@med.uoa.gr; 4 Laboratory of Medical Microbiology, Department of Microbiology, Hellenic Pasteur Institute, 11521 Athens, Greece

**Keywords:** hepatitis B virus, HBV, viral integration, pathogen-host interactions, insertional mutagenesis, chronic hepatitis, Hepatocellular Carcinoma

## Abstract

Hepatitis B Virus (HBV) is an Old World virus with a high mutation rate, which puts its origins in Africa alongside the origins of Homo sapiens, and is a member of the Hepadnaviridae family that is characterized by a unique viral replication cycle. It targets human hepatocytes and can lead to chronic HBV infection either after acute infection via horizontal transmission usually during infancy or childhood or via maternal–fetal transmission. HBV has been found in ~85% of HBV-related Hepatocellular Carcinomas (HCC), and it can integrate the whole or part of its genome into the host genomic DNA. The molecular mechanisms involved in the HBV DNA integration is not yet clear; thus, multiple models have been described with respect to either the relaxed-circular DNA (rcDNA) or the double-stranded linear DNA (dslDNA) of HBV. Various genes have been found to be affected by HBV DNA integration, including cell-proliferation-related genes, oncogenes and long non-coding RNA genes (lincRNAs). The present review summarizes the advances in the research of HBV DNA integration, focusing on the evolutionary and molecular side of the integration events along with the arising clinical aspects in the light of WHO’s commitment to eliminate HBV and viral hepatitis by 2030.

## 1. Introduction

In 1965, Dr. Baruch Blumberg and his colleagues, during studies completely unrelated to viral hepatitis, discovered the “Australia antigen” [1], which was afterwards identified as the hepatitis B virus surface antigen (HBsAg). This discovery was awarded with the Nobel Prize in Physiology or Medicine in 1976 and is often believed to mark the beginning of hepatitis research [2]. In fact, the research on the pathogenesis of viral hepatitis had started decades before Dr. Blumberg’s critical breakthrough. In 1865, Rudolf Carl Virchow, one of the most prominent physicians of the 19th century, provided an authoritative explanation of the transmission by human serum of the “catarrhal jaundice” [3], which had been described by Hippocrates [4].

In the early 1920s, the distinction between “infectious” and “serum” hepatitis was recognized [2] and in 1947, the terms “hepatitis A” and “hepatitis B” were introduced [5]. Chronic hepatitis was first reported in the 1940s after observations of patients with cirrhosis, who had no history of alcoholic liver disease, but who had recovered many years earlier from an episode of catarrhal jaundice [6], and the development of liver function tests led to the recognition of anicteric infections and the existence of chronic carriers in the 1950s.

After the discovery of the “Australia antigen”, the vast amount of accumulated epidemiological and clinical data, as well as the huge numbers of stored serum samples, enabled rapid progress in understanding hepatitis B, and revealed the existence of a vast population of chronically infected people in multiple regions of the world [2]. After all these critical breakthroughs, HBV-related studies grew in number. In 1972, the first vaccine against HBV was available [7], in 1986, the first clinical trial of interferon—a therapy of chronic hepatitis B—started [8], and, in 1998, the first direct-acting antiviral (lamivudine) for chronic hepatitis B was approved by the FDA [9].

The identification of integrated HBV DNA in human genomic DNA was first reported in the early 1980s from four separate research groups [10,11,12,13], while more research studies were subsequently carried out to further examine the HBV DNA integration process. Most of the HBV integrations were not found to be recurrent, as each integration site was identified in only one sample [14,15,16,17]. In the early 2000s, the first recurrent HBV DNA integration site was identified in humans *TERT* gene [18,19] and from them on more recurrent HBV DNA integration sites have been reported from different research groups, using multiple methodologies.

**Figure 1:**
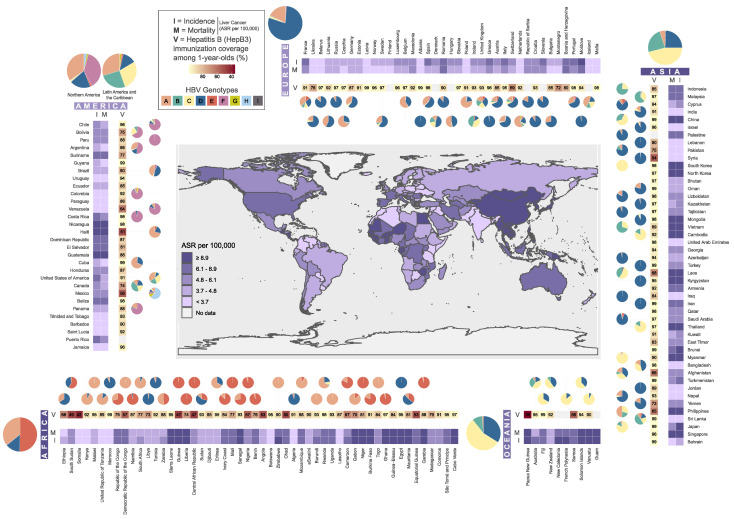
Geographical distribution of liver cancer incidence and mortality rates (Age-Standarized Rate-ASR per 100,000—data derived from WHO-Global Cancer Observatory: Available online: gco.iarc.fr (accessed on 8 May 2012)), percentage of Hepatitis B (HepB3) immunization coverage among one-years-olds (data derived from WHO–Global Health Observatory data repository: apps.who.int/gho/data/view.main.80300 accessed on 18 August 2021) and HBV genotypes (data derived from Velkov et al. [36]). The world map has been colored with respect to the Liver Cancer Incidence Rate (ASR per 100,000). The ASR Incidence and Mortality as well as the percentage of the HepB3 immunization coverage among one-years-olds are depicted as a heatmap, while the pie charts represent the proportional (%) HBV genotype distributions in the respective countries. All plots were generated using the ggplot2 R package [37].

Despite the availability of a vaccine since the 1980s, HBV remains a global public health problem, with an estimated 257 million people with chronic HBV infection, 90% of whom are unaware of their infection [20,21] and, as a result, do not receive appropriate medical care. Moreover, in many low-income and remote regions worldwide, vaccine coverage is another barrier in the disease control, and Africa and the Western Pacific region are disproportionately affected by HBV (Figure 1), (www.who.int/publications/i/item/global-hepatitis-report-2017 accessed on 18 August 2021).

The total population living with chronic HBV infection is indicative of the historical prevalence of HBV, whereas the prevalence among children aged 5 years also reflects access to preventive strategies, particularly infant vaccination [20]. For example, most of the countries of the SEARO (South East Asia) regions have accepted vaccination against HBV in their EPI program (Expanded Program on Immunization), while in China—which has the greatest number of HBsAg-positive individuals—had a percentage of 99% HBV immunization coverage among one-year-olds in 2019 (Figure 1), (WHO Global Health Observatory data repository: apps.who.int/gho/data/view.main.80300 accessed on 18 August 2021).

Simultaneously with the continuation of mass vaccination against HBV, other challenges must be overcome, including the prevention of mother to child HBV transmission by the timely administration of the HBV birth dose vaccine (within 24 h of birth) [22], the improvement of injection safety, the routine screening of all blood donations for transfusion, as well as the provision of sterilized syringes to people who inject drugs (PWID) (WHO-Global Health Sector Strategy on Viral Hepatitis 2016–2021: apps.who.int/iris/bitstream/handle/10665/246177/WHO-HIV-2016.06-eng.pdf accessed on 18 August 2021). Moreover, the conduction of nation-wide and workplace-based [23] surveys on HBV prevalence, mortality, and genotype distribution will provide more insights and feedback on the HBV prevention and elimination process.

## 2. Evolution of HBV and Co-Existence with Human Populations

HBV is a small spherical virus with icosahedral symmetry that is classified under the Hepadnaviridae family. The virus combines a partial double-stranded DNA (ds-DNA) and virus-encoded RT, and consequently it is classified as Group VII in Baltimore’s classification system (also referred to as pararetroviruses). The Hepadnaviridae family is characterized by a unique viral replication cycle, as the virus-encoded polymerase has reverse transcriptase (RT), DNA-polymerase (pol), and protein priming activities, while the reverse transcription step takes place in the late stages of the genomic replication [24]. Moreover, the polymerase lacks proofreading activity and, as a result, creates genetic variability that is manifested as circulating viral quasi-species [25].

The HBV substitution rate is estimated as ~1.5–3.0 ×$10^{-6}$ substitutions per site per year, while the origin of the virus is estimated at 33,600–34,100 years ago [26,27]. Chronic infections, including extended asymptomatic periods and a slow progression to clinical disease—characteristics that are also observed in Mycobacterium tuberculosis [28] and Helicobacter pylori [29] infections—are features suggesting that HBV co-existed with human populations for many thousands of years [25].

HBV sequences have been isolated from 400-year-old mummies from Korea [30] and Italy [31], while the analysis of ancient DNA derived from 7000-year-old skeletal remains from across Eurasia gained more insights about the origin and age of HBV [32,33]. Recent descriptions of members of the Hepadnaviridae family isolated from fish, reptiles and amphibians [34] strengthened the HBV—host species co-evolution theory [35].

## 3. Geographical Distribution of Human HBV Genotypes and Their Role in the Natural History of the Infection

There are nine confirmed (HBV A-I) and one putative (HBV J) human HBV genotypes, while some of them are further subdivided into sub-genotypes (over 30 in number) with particular global distribution patterns [38], disease and clinical progression, response to antiviral treatment and prognosis. HBV genotypes are characterized by a >8% nucleotide difference, while sub-genotypes are characterized by 4–8% nucleotide differences [39]. Furthermore, the geographical distribution of HBV genotypes is possibly related to the route of transmission and whether the exposure to the virus is perinatal, vertical or horizontal.

As presented in Figure 1, genotype A is widespread in Africa, Southern Asia, Europe and North America, and it is related to early childhood transmission, while genotypes B, C and E are localized in high-endemic regions (SE Asia, Western and Central Africa, China, Japan and Australia) and have been related to maternal–fetal, perinatal exposure. Genotype D is distributed globally and has been associated with early childhood or adult horizontal transmission [39], while genotypes F–H are localized mainly in the USA. Finally, the recently identified genotypes I and J are localized in Asia and specifically in Laos, Vietnam, China (genotype I) and Japan (genotype J), respectively.

The HBV genotype distribution is heterogeneous, and there is a lack of accurate genotyping data and monitoring of the epidemic genotypes. This makes epidemiological studies reflect only snapshots of the epidemiology while different HBV genotypes have different dynamics and may drift and change dynamically over time [40]. For example, although genotypes B, C and E have been reported to be prevalent in Asian countries, recent studies have reported that Bangladeshi patients have three major HBV genotypes (A, C and D) in considerable proportions [41].

Specifically, Raihan et al. [41], found that there was an association between HBV genotype C and high levels of HBV DNA and ALT, liver cirrhosis and HCC, while genotypes D and A are prevalent in patients with persistently low HBV DNA and normal ALT levels. Absence of the HBV genotype B has been reported from India, as well [42]. Furthermore, a recent study conducted by Velkov et al. [36] regarding the global HBV genotype distribution underlined that, in Latin America the genotypes F, G and H—that are rare in other parts of the world—were found in significant proportions. Moreover, they revealed the domination of genotypes A and D in Brazil as well as the existence of genotypes G and H in Mexico that are not present in other populations (Figure 1).

## 4. Natural History of Chronic HBV Infection

The natural history of human HBV infection varies between the different genotypes [25], as the predominant mode of transmission, the timing of the Hepatitis B e antigen (HBeAg) seroconversion to the hepatitis B e antibody (anti-HBe), the progression of liver-associated diseases—including Hepatocellular Carcinoma (HCC), and the potential of Hepatitis B surface antigen (HBsAg) seroclearance, vary substantially Box 1 [38]. Specifically, patients infected with the genotype C remain HBeAg-positive for many years longer than patients infected with any of the genotypes A, B, D or F [43].

Box 1 **Serological markers of HBV infection.**

**HBeAg (Hepatitis B e antigen):** Protein molecule contained in the nucleocapsid core of HBV that is detected in the serum of persons with high virus titers indicating high infectivity.

**HBcAg (Hepatitis B core antigen):** Protein molecule on the surface of the nucleocapsid core that is not secreted and, as a result, cannot be detected in the serum of infected individuals (however, its antibody can). Its presence indicates ongoing HBV replication during active infection.

* HBeAg and HBcAg originate from the same ORF at the HBV genome.

**HBsAg (Hepatitis B surface antigen):** Protein molecule on the surface of HBV, which can be detected in high levels in serum during acute or chronic HBV infection and indicates that the person is infectious. HBsAg is the antigen used to make the vaccine against HBV.

**anti-HBs (Hepatitis B surface antibody):** The presence of anti-HBs indicates recovery and immunity from HBV infection as well as successful vaccination against HBV.

**anti-HBc (Total hepatitis B core antibody):** Appears at the onset of symptoms in acute hepatitis B and persists for life, indicating previous or ongoing HBV infection in an undefined time frame.

**IgM anti-HBc (IgM antibody to hepatitis B core antigen):** Positively indicates recent (≤6 months) acute HBV infection.

The risk of developing chronic HBV infection depends on the age of exposure to HBV. Acute HBV infection within the first year of life has a 90% probability; in childhood, 20–30% probability; and in adulthood, a <5% probability of progression to chronic HBV infection [44]. Moreover, most of the neonatal and perinatal HBV infections become persistent [45]. The fact that, in early stages of the life cycle, HBV infections lead in such great percentages to chronic HBV infection is due to immune inhibitory mechanisms that either delete the HBV-specific T-cells in the thymus or render them dysfunctional in the periphery [46].

Chronic HBV infection is a complex and dynamic condition, as infected individuals can move from a phase of high viral load without liver disease to a phase of active liver disease, followed by a phase of inactivation and revert back to active liver disease [47]. Not all individuals with chronic HBV infection develop cirrhosis, as clinic-based longitudinal studies suggest that the overall incidence is 2–3% per year [48], while factors, such as the older age, the long-term presence of HBeAg and elevated levels of alanine aminotransferase (ALT) increase the risk [49].

**Figure 2:**
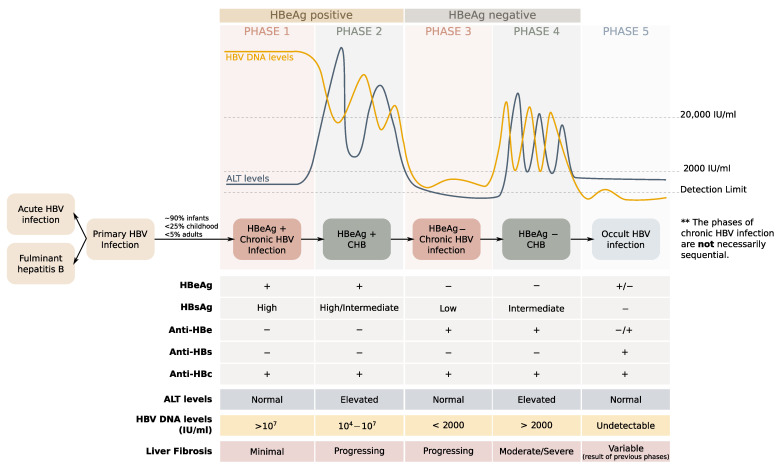
The natural history of chronic hepatitis B is described by five phases—which are not necessarily sequential: the HBeAg positive chronic HBV infection or immune tolerant phase (phase 1), the HBeAg positive chronic hepatitis or immune reactive HBeAg positive phase (phase 2), the HBeAg chronic infection or inactive carrier phase (phase 3), the HBeAg negative or HBeAg negative chronic hepatitis (phase 4) and the Occult HBV infection or HBsAg negative phase (phase 5) [50]. During Phase 1, the HBV DNA levels are very high (>$10^{7}$ IU/mL), and HBV integrates parts or the whole of its genome into the host’s hepatocyte genomic DNA as a result of the reverse transcription mediated by the HBV polymerase. The alanine transaminase (ALT) levels are within the normal range, and there is minimal or no liver necroinflammation or fibrosis. Phase 2 is described by high levels of HBV DNA and elevated ALT levels, while there is moderate fibrosis that is accelerating. From this phase most patients transit to Phase 3 after HBeAg seroconversion, while others progress to Phase 4 for many years. Phase 3 is characterized by low (<2000 IU/mL) HBV DNA levels and normal ALT levels. Patients who remain in this phase have a low risk of progression to cirrhosis or HCC but progression to CHB and transition to Phase 4 usually occurs. Phase 4 is described by high levels of serum HBV DNA and elevated ALT levels, while liver necroinflammation and fibrosis are present. Phase 5 is characterized by undetectable levels of HBV DNA and normal ALT levels, while the liver histology and the risk of HCC are sequelae of the previous phases. The general shape of the curves that describe HBV DNA and ALT levels has beeen adapted from [57].

There are five phases of chronic HBV infection: the immune tolerant phase, two immune active phases, the inactive chronic HBV infection phase and the recovery phase (Figure 2) [50]. The immune tolerant phase or HBeAg-positive chronic HBV phase (phase 1) lasts longer in patients infected via maternal–fetal transmission from HBeAg-positive mothers [43], which occurs more frequently in HBV genotype C infections. It can last for a few months or typically from years to even lifelong [51], the levels of ALT are normal, and there is either no or minimal liver inflammation or fibrosis.

During this phase, HBV integrates parts or whole of its genome into the host’s hepatocyte genomic DNA, as a result of the reverse transcription mediated by the HBV polymerase, and high levels of serum HBV DNA (>1 million DNA copies, >200,000 IU/mL) are present. During the HBeAg-positive immune active phase or HBeAg-positive chronic hepatitis B (CHB) (phase 2), inflammatory reaction occurs as a result of the host’s immune response, elevated ALT levels are observed and serum HBV DNA levels progressively decrease but remain high (>20,000 IU/mL). Active liver inflammation is usually present with or without significant liver fibrosis [47].

After the seroconversion from HBeAg to anti-HBe, most of the infected individuals (>80% of the cases) will transition into the HBeAg-negative inactive chronic infection phase, while the others will directly progress in the HBeAg-negative immune active phase or HBeAg-negative CHB [52]. The inactive chronic HBV infection or carrier phase (phase 3), is characterized by HBeAg seroclearance and anti-HBe positivity. The ALT levels are within the reference range, the HBV DNA levels are low (usually <2000 IU/mL), the liver inflammation recedes, and HBsAg is still present. This is also the phase when multiple integration events occur within the host’s genome.

The inactive HBV carrier phase may last the lifetime, while some patients may reverse to HBeAg seropositivity (usually within the first 12 months after HBeAg seroclearance) or a proportion of cases (20–30%) may develop HBeAg-negative CHB (phase 4). Except for the presence of HBeAg in serum, the characteristics of the immune active phase of HBeAg-negative CHB are similar to those of HBeAg-positive CHB, although the median serum HBV DNA levels are relatively lower. Finally, during the recovery phase (phase 5), neither HBV DNA nor HBsAg can be detected into the serum, while various antibodies have been produced against HBV (anti-HBs and anti-HBc) [52,53].

Gish et al. [54] proposed two more theoretical clearance phases; the clearance of cccDNA and the clearance of cells that have integrated HBV. These two phases have been extensively studied recently, as they are considered key points for the development of novel future therapies against HBV. The complete clearance of cccDNA in vivo is rare, while two immune mechanisms have been proposed. Based on the cytolytic mechanism, infected cells are killed by cytotoxic T lymphocytes and are being replaced by uninfected hepatocytes, while, based on the non-cytolytic cytokine-induced mechanism, the clearance is achieved through the activation of intracellular antiviral pathways [55].

In general, it is considered that clearance of HBV is performed via HBV-specific CD8+ T cells that escape central tolerance and attack the infected hepatocytes, while a small proportion of HBV-specific CD8+ T cells without the ability to produce antiviral cytokines possibly sustain long-term immunopathological responses and whether they manage to achieve HBV clearance is unknown [56]. The reverberation of HBV integrations in this process is an issue that needs to be explored as there is no reported evidence.

## 5. Genome Organization and Types of Genomic Material within the HBV Life Cycle

The HBV life cycle is a process with numerous steps and increased complexity that arises from the different types of HBV particles observed in the serum of infected patients, the different states in which HBV genomic DNA is found in the viral particles, as well as the HBV genome, itself, with the overlapping open reading frames (ORFs).

Four categories of HBV particles have been sufficiently described in the serum of infected patients; the Dane particles, the enveloped (nucleo)capsids containing immature DNA/RNA or as genome-free (empty virions), the subviral particles (SVPs) and the filament structures of variable length [58,59]. A fifth type is the envelope-less, naked nucleocapsids; however, this type has been described only in vitro [58]. From these, only the 42 nm—diameter spherical Dane particles are infectious, while the 22 nm—diameter enveloped (nucleo)capsids and SVPs are non-infectious but much more abundant in the patient serum.

The Dane particles [60], that were named by the scientist who first visualized them using an electron microscope in 1970, consist of a lipid membrane with three HBV surface antigens (HBs) with different sizes: large (L-HBs), middle (M-HBs), and small (S-HBs). The lipid membrane surrounds the nucleocapsid (composed of the HBc), the HBV polymerase (Pol) and the genomic DNA. Both the enveloped and the naked nucleocapsids may contain DNA, RNA or no genomic material [59]. Moreover, the genome-free enveloped capsids (empty virions) have been recently suggested as diagnostic markers for hepatic cccDNA [61] as well as candidates for a new generation of HBV vaccine [58].

**Figure 3:**
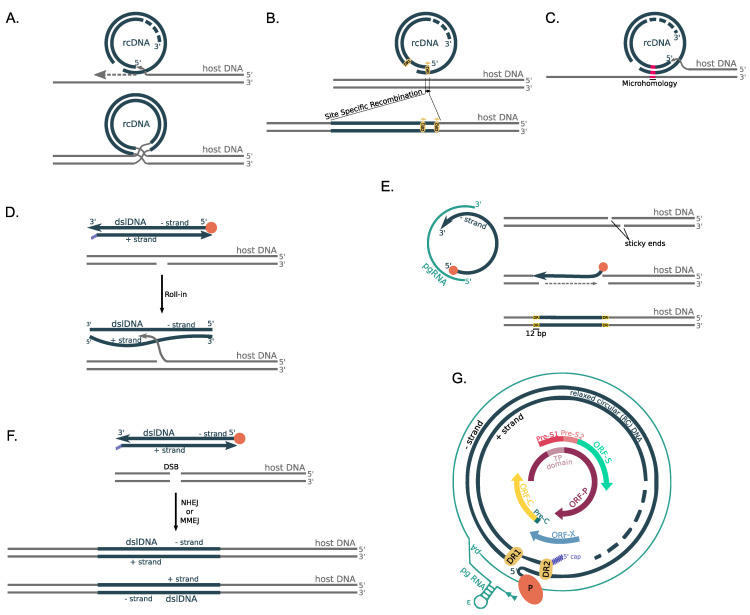
The molecular mechanisms of HBV DNA integration into the host genome with respect to the relaxed circular DNA (rcDNA) and double-stranded linear DNA (dslDNA) structure. (**A**) The ‘single-stranded-gap’ model [11], implies that the DNA polymerase switches from the human genomic DNA strand to the 5′ single-stranded region of the nearby HBV genome, followed by a recombination event. (**B**) The HBV ‘DR-mediated integration’ model [86] implies that HBV DNA integration occurs via a site-specific recombination event between host genomic DNA and either in DR1 or DR2. (**C**) The ‘partial-sequence homology’ model [90] implies that a microhomology between the host DNA and the HBV genome triggers the switch of the DNA polymerase from the host DNA to the single-stranded region of the HBV rcDNA, which is followed by a recombination event in the site of the microhomology that determines the site of integration. (**D**) The ‘roll-in’ model [14] is based on HBV dslDNA where the free end at a single-strand break in cellular DNA invades into the HBV genome and forms the host–viral junction. (**E**) The minus strand of the HBV rcDNA is released from the replication complex before HBcAg synthesis, while a staggered break at the host DNA leaves sticky ends in which the incomplete HBV genome is inserted, resulting in the formation of 12 bp DRs that are neither viral nor cellular on either side of the viral integration site [93]. (**F**) The ‘NNEJ/MMEJ-based model’ implies that the HBV dslDNA is integrated at a site of DSB after its repair and removal of the Pol protein and the 5’cap via Non-Homologous End Joining in both directions or via Microhomology-Mediated End Joining in a direction determined based on the microhomology. (**G**) The relaxed-circular HBV DNA structure and genome organization. The inner region depicts the organization of the HBV genes, the dashed blue circle represents the (+) strand, the solid blue circle represents the (−) strand and the outer petrol-blue line represents the HBV pgRNA.

The HBV genome is mainly composed of overlapping ORFs (Figure 3G) from which multiple functional proteins are produced. HBc, HBeAg and 22-kDa precore protein (p22cr) are produced from *ORF-C*; Pol is produced from *ORF-P*; L-HBs, M-HBs and S-HBs are produced from *ORF-S*; while the HBV X protein is produced from *ORF-X*.

During the life cycle of the virus, the HBV genomic DNA, can be found in four different states, two of which are candidates for HBV integration into the host genome. After the glycosaminoglycan-mediated viral entry—through the sodium taurocholate cotransporting polypeptide (NTCP) receptor [62], which is located in the basolateral membrane, into the hepatocytes, nucleocapsids enter the cytoplasm and are directed to the nucleus along with the microtubules [63,64]. At this stage, the nucleocapsids, contain relaxed circular DNA (rcDNA) or, more rarely, double-stranded linear DNA (dslDNA).

In the nucleus, the rcDNA is modified by cellular factors, including Tyrosyl DNA Phosphodiesterase-2 (TDP2) [65,66], DNA polymerase kappa (Pol kappa) [67], Pol alpha [68], DNA ligase 1 (LIG1) and LIG3 [69] and flap endonuclase 1 (FEN1) [70]. Specifically, the Pol-linked terminal redundant sequence in the 5${}^{\prime }$ end of the minus strand DNA and the RNA oligonucleotide attached at the 5${}^{\prime }$ end of the plus strand DNA are removed from the rcDNA [59,71,72].

The gaps in both strands are filled and ligated to generate covalently closed circular DNA (cccDNA), which is a stable episomal transcriptional template for the HBV mRNAs [73]. The cccDNA is transcribed into five HBV RNAs of different length, under the action of the host’s RNA polymerase II [74]. One of the five transcripts is the 3.5 kb length pre-genomic RNA (pgRNA). The pgRNA can be encapsidated into viral capsids, in a process mediated by the HBV polymerase [64]. In the nucleocapsids, reverse transcription of the pgRNA results in the formation of rcDNA or dslDNA.

The intranuclear HBV dslDNA is the main candidate of DNA molecule that can be integrated into the host genome. It has been shown that the integration occurs at a frequency of 1 in 10${}^{2}$ to 10${}^{4}$ cells in the woodchuck and duck models of HBV infection [75,76,77] and in chronically infected HBV patients [78,79,80]. Based on cell-culture experiments, the integration of HBV DNA into the host genome occurs within a week after the infection [81] via double-stranded DNA breaks by nonhomologous end joining (NHEJ) or micro-homology mediated end joining (MMEJ), as discussed below.

However, a series of NGS-based studies [82,83,84] reported that some of the identified HBV integration sites are uncorrelated to the dslDNA termini; thus, other HBV DNA forms may be important contributors in the integration process as well. The cohesive-end linear DNA, that occurs via denaturation of the 5′ cohesive overlap region of HBV rcDNA and subsequent extension of the recessed 3′ ends and formation of terminal redundancies between the direct repeat 1 (DR1) and DR2 as well as spliced variants of HBV RNA, may be the missing key points in the deciphering of the HBV integration process into the host genome [85].

## 6. Proposed Molecular Mechanisms of HBV DNA Integration

A series of molecular mechanisms have been described for the integration of HBV DNA into the human genome (Figure 3). All of the above-mentioned molecular mechanisms were proposed after thorough examination of the virus-host junction sites and the effect of the integration in the cellular DNA, which can be from minimal to disruptive.

The “single-stranded gap” model (Figure 3A) is based on the restriction maps and nucleotide sequences of the human-HBV DNA junctions, and it indicates that, during cellular DNA replication, the DNA polymerase switches from the human genomic DNA strand to the single-stranded region of nearby HBV genome [11]. This switch is restricted to the gap region close to the 5${}^{\prime }$ end as procession of another DNA polymerase complex starting from the 3${}^{\prime }$ end of the short strand of HBV limits the available single-stranded region. A recombination event then occurs and joins the long strand of the HBV dsDNA with the original human genomic DNA and after filling in and ligation, a full-length HBV integration site is present in the human genome.

Two more models were described after the observation that a 11 bp length sequence of the host–viral junctions matches the Direct Repeat (DR) sequences that are located close to the cohesive-end region of the HBV sequence [86]. Both of the models are based on the observation that integration of the DR sequence into the host genome occurs after deletion of the first two nucleotides of the DR sequence, the conservation of the DR1/DR2 polarity and the presence of viral-specific and non-specific sites of integration. The first of the two models assumes that one of the DRs is used as the target site for the formation of an initial host–viral junction via site-specific recombination, which is followed by a second recombination event, which forms the integration site (Figure 3B).

The second model that includes the DR sequences is based on the assumption that HBV DNA is re-organized prior to integration, forming ‘head-to-head’ structures that give rise to inverted direct repeats [86]. HBV integration is mediated by two inverted DR sequences [75,77,78,82,83,84,87,88,89]. Inverted DRs are known to be unstable, and thus deletions within the HBV DNA are expected to occur, leading to further re-organization of the HBV DNA that possibly results in the loss of at least one DR sequence.

Another proposed mechanism is based on the partial sequence homology between the HBV DNA and the host genomic DNA at the site of the integration (Figure 3C) [90], as it has been found that integrations result in the termination of transcription at a cellular termination signal. Based on this molecular mechanism, the integrations result in the expression of viral–human chimeric fusion proteins that potentially contribute to cancer progression as will be discussed below.

The next model is based on the idea of a site-specific mechanism of recombination [91,92], in which the 3′ free end of the minus HBV strand facilitates strand invasion into a double-stranded break or gap that is formed by the HBV infection or endogenous metabolism at the 5′ end of the host DNA in a process that leads to a temporary base pairing between the two DNA molecules because of a short homology region (Figure 3D). This process has been described as a “roll-in” method [14], and it leads to full or partial HBV DNA integration.

Another proposed mechanism is based on the observation that integrated HBV DNA into the human genome is flanked on both sides by a 12 bp repeated sequence that is neither viral nor cellular but has been generated due to the integration itself [93]. Termination of the reverse transcription of the pgRNA before the completion of the transcription of the minus strand results in the formation of an incomplete minus strand with the absence of the HBcAg.

The 3′ end of the minus strand is joined to the 5′ overhang at the site of a staggered cut, the covalently bound viral DNA polymerase at the 5′ end of the minus strand is removed and the staggered ends are filled in and ligated creating two equal-sized direct repeats of the human flanking sequence (Figure 3E). In addition to other proposed models, this particular model assumes that there is no deletion or loss of human genomic sequences at the site of the integration.

The last method is based on the non-homologous end joining (NHEJ) molecular mechanism mediated by cellular enzymes and, in particular the topoisomerase I, leading to a 11 bp deletion of the host genome [94,95]. This mechanism takes into account the absence of sequence homology between the HBV and host DNA at the majority of the virus-host junctions [81,84]. Due to the microhomology that has been described in some of the junctions, the microhomology-mediated end joining (MMEJ) has also been described as a proposed mechanism of HBV DNA integration (Figure 3F) [81,83,96,97,98].

Finally, a major key point that should be taken into account in order to better understand the molecular mechanisms that describe the HBV integration process and, in general. the process of viral integrations into genomes, is the fact that the immediate integration events that occur after HBV infection are followed by additional post-integration changes due to genomic instability at the sites of integration. Moreover, integrations that occur within transposable and retrotransposable elements are very important since the transposition of these elements increase the “pool” of integrations, while the capacity of the integrated HBV to be excised from one genomic loci and transported to another genomic loci through reintegration [99] should be further investigated.

## 7. Hepatocellular Carcinoma (HCC)

HCC is the most common subtype of liver cancer and the forth leading cause of cancer death worldwide. Specifically, it has been estimated that HCC is the fifth most common cancer in men and the ninth in women, with approximately 500,000 and 200,000 new cases per year worldwide, respectively. As for the global distribution of HCC, 76% of the annual reported cases originate from Asia, 8.1% from Europe, 7.5% from Africa, 4.2% from North America, 3.8% from Latin America and the Caribbean and 0.4% from Oceania (Figure 1)(WHO data: www.gco.iarc.fr—Accessed on 8 June 2021).

The combination of the host genetic background and the access to health care and prevention facilities has an influence on the outcome of the disease, as belonging to a specific population group increases the susceptibility of HBV carrier to develop HCC [100]. Specifically, mortality due to HCC is increased in African and Asian populations (WHO data: www.gco.iarc.fr—Accessed on 8 June 2021), and, at the same time in these two populations, more than 70% of HCC develop in cases with HBV infection [101].

HBV infection causes chronic hepatitis and, subsequently, liver cirrhosis, while it has been identified as one of the most important contributors of HCC, as ~50% of the cases are due to HBV infection [102]. Specifically, it has been estimated that the development of HCC is 25–37-fold higher in carriers of HBsAg compared with non-infected individuals [103,104].

Research suggested that approximately 80% of tumor derived tissues are carrying integrated HBV DNA [15,105,106]. Adjacent tissues also harbor HBV integration events [107], and it has been proposed that viral integration occurs during chronic HBV infection, before HCC initiation [15,105]. Specifically, it has been suggested that hepatocytes with HBV integration undergo certain rounds of expansion during chronic hepatitis and some of them participate in the formation of focal proliferative lesions. As a result, they gain growth advantages, and they clonally expand during tumorigenesis [107].

In general, HBV integration is more frequent in tumor samples than in adjacent liver tissues, at percentages of 86.4% and 30.7%, respectively [82], while there is no reported difference in the chromosome distribution between tumor- and adjacent non-tumor-derived samples [105]. Alu-PCR, NGS and in silico approaches revealed that there is a preference for HBV integration to chromosomes 3 [108], 11 [107,109] and 17 [109].

## 8. HBV Integration Sites into the Human Genome and Target Genes

HBV integration into human host chromosomes occurs in the infected hepatocytes since early stages of natural acute infections [15,100,105]. Specifically, it has been shown that integration starts <3 days after the infection in cell cultures [81] and in the early stages of infection in vivo [75,110]. Deletions, duplications, inversions, copy number variations (CNVs) and other types of rearrangements of the HBV sequence have been observed in the sites of HBV integration, while sequencing analysis of the viral-host junctions revealed only a few micro-deletions, micro-insertions, point mutations and translocations with no significant difference being found between tumor-derived and non-tumor-derived samples [82,105].

HBV integrations induce chromosome changes, genome instability and changes in the expression of host genes, while they have been associated with chromosome fragile sites or repetitive sequences, followed by local rearrangements, which further increase the genomic instability [111]. It should also be taken into account that HBV integrations have the potential to endlessly continue during persistent and chronic infections [56], and thus the number of HBV integration sites per sample may be associated with the time of sample collection that reflects the phase of the HBV infection.

**Figure 4:**
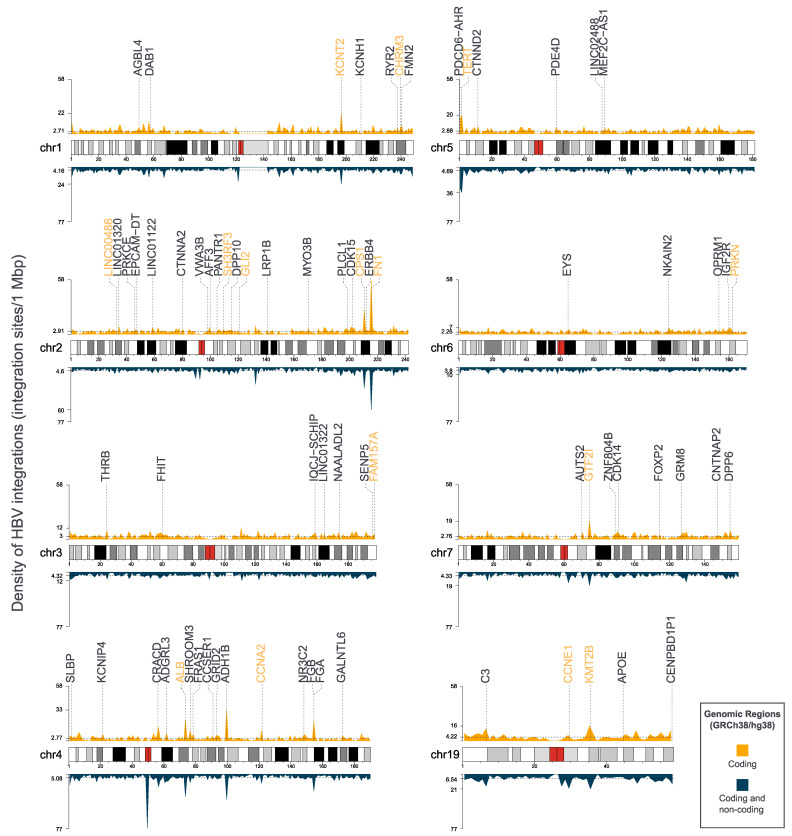
HBV DNA integration density plots for the chromosomes 1–7 and 19 in which the recurrently affected genes are localized. The yellow density plots represent the density of integrations per 1 Mbp (1,000,000 bp) through the chromosome that are referring to coding genomic regions, while the blue density plots represent the density of integrations per 1 Mbp that are referring to all genomic regions (coding and non-coding). The mean values are depicted via the horizontal dashed lines, and the locations of the main target genes are also present. The plots were generated with the karyoploteR R package [187].

More than 10,000 unique HBV integration sites into the Human Genome have been reported in 25 different research studies, and ~51% (6864/13,572) of which have been found to target genomic regions that refer to 4183 different genes. It has been suggested that genomic areas that refer to cellular genes (Figure 4) are favored target sites for HBV integration [105,112], and specifically there is a preference of HBV integration into transcriptional and chromosomal regulatory regions [113]. DR1 and the topoisomerase I motif are the preferred break-points of the inserted viral fragments in both orientations [105]. Moreover, 2602 integration sites are recurrent (~19.2%), or, in other words, they have been reported more than once in different cases from independent research groups.

The main target genes of HBV integrations are the *TERT, FN1, KMT2B, ALB, CCNA2, LINC00486, CPS1, SH3RF3, CCNE1, GLI2, KCNT2, LINGO2, PRKN, FAM157A, SOX5, GTF2I, PDE3A* and *CHRM3* genes (Supplementary Figure S1), that have been reported in more than twenty studied cases as presented in Table 1 and Figure 4.

### 8.1. Genes Involved in the Cell Cycle G1/S Transition (Gene Ontology (GO) ID:0044843)

The accurate transition from G1 to S phase of the cell cycle is critical for the control of the eukaryotic cell proliferation, while it has been found that misregulation promotes oncogenesis [131]. The *CCNA2*, *CCNE1* and *TERT* genes take part in the G1/S transition process, and they have been found to be major genomic locations of recurrent HBV DNA integrations.

The proteins encoded by the *CCNA2* and *CCNE1* genes, cyclin A2 and cyclin E1, respectively, belong to the highly conserved cyclin family. All the members of the cyclin family are characterized by a dramatic periodicity in protein abundance through the cell cycle. Both proteins regulate the cell cycle by promoting the S phase entry and progression. Specifically, the cyclin A2 promotes the transition through G1/S and G2/M via activation of the cyclin-dependent kinase 2 while cyclin E1 accumulates at the G1/S phase boundary and is degraded as cells progress through S phase. Both genes are disrupted by HBV DNA integrations more frequently in tumors than in non-tumor samples. Moreover, cyclin A2 and E1 alterations define a homogenous entity of aggressive HCC, which is mostly developed in non-cirrhotic patients [130].

Cyclin-driven HCC displays a unique signature of structural rearrangements with hundreds of tandem duplications and templated insertions frequently activating the *TERT* promoter [130]. The *HBx*/*HBsAg* genes are the parts of the HBV genome that are mainly integrated into the *CCNE1* [127,132,133]. It has been proposed that the disregulation of cyclins and especially interruption of the *CCNA2* gene plays a direct role in cellular transformation and oncogenesis through the loss of cell cycle control [118,134]. As for the gene expression values, researchers suggested that up-regulation of cyclin-encoded genes results in chromosome instability and contributes to cancer progression, while it has been found that HBV integrations—particularly in the promoter region—elevate the gene expression levels [124].

The *TERT* gene is a protein-coding gene that contributes in the formation of the telomerase enzyme whose role is the maintenance of the telomeres. Telomeres role is the protection of chromosomes from abnormal sticking together and degradation. In most cells, including the hepatocytes, telomeres are progressively shortened because of cell division, and, when they reach a critical length, they trigger the cell to stop division and undergo apoptosis. The role of the telomerase is crucial in this process as it adds small repeated segments of DNA to the ends of the chromosomes each time the cell divides.

In general, the expression levels of the *TERT* gene are either undetectable or very low. Abnormal activation of telomerase has been reported in various types of cancers (including mostly melanoma and acute myeloid leukemia) and leads to cell growth and division without order. *TERT* gene disruptions have been associated with HCC, and particularly HBV integration in the *TERT* locus possibly confers a clonal advantage in the early phase of HBV-related liver carcinogenesis [114].

Over-expression of the *TERT* gene has been reported due to HBV DNA integration [82,107,121,129], while the possible functional impact of the HBs-*TERT* chimera that has been reported to occur after integration, should be further investigated [118]. Moreover, the gene disruption mediated by HBV integration can cause uncontrolled cell growth and can be a contributing factor in the formation of HCC in patients with chronic HBV infections.

Studies have found that the integration is more frequent in the promoter region than in the intronic region of the *TERT* gene [107,116,121,125,135] and that this possibly results in a “cis” effect on the transcription of the gene [17,82,125]. It has been also suggested that the HBV-*TERT* integration frequency may differ in different populations and HBV subtypes [127].

### 8.2. Genes Involved in the DNA Replication (GO:0006260)

In addition to the *CCNA2*, *CCNE1* genes, the *GLI2* gene is also involved in DNA replication, and it has been found to be affected by HBV integrations. The *GLI2* gene encodes a protein that belongs to the Gii family and specifically to the C2H2-type zinc finger protein subclass, which has been characterized as a transcription factior (TF) with the ability to bind DNA through zinc finger motifs. The genes of this subclass have been described as potential oncogenes in the embryonal carcinoma cell while the *GLI2* gene possibly plays a role during embryogenesis. Moreover, *GLI2* is considered a marker of activation of the sonic hedgehog signaling pathway, which has been associated with aggressive types of renal cell carcinoma [136,137,138] as well as HCC [139,140].

HBV DNA integrations within the *GLI2* gene are more frequent in tumor samples, while there is no reported evidence regarding the gene expression values in hepatocytes with HBV integrations. Studies regarding the expression of *GLI2* in HCC tissues have revealed that it is up-regulated [139,141], and it has been associated with a poor prognosis in patients with HCC after hepatectomy [141].

### 8.3. Genes Involved in the Histone Modification (GO:0016570)

The *KMT2B* gene takes part in the histone modification process along with the *CCNA2* gene. The *KMT2B* is an oncogene [142] that encodes the lysine-specific methyltransferase 2D enzyme that is found in many organs and tissues, including the liver, while its main role is the regulation of developmental-related genes. Moreover, it has been described as a tumor suppressor gene as it helps in the prevention of uncontrolled cell growing and division.

HBV integration sites in the *KMT2B* gene are more frequent in tumor samples, while it has been reported that the gene is upregulated in tumor samples relative to the adjacent normal tissue samples [82]. Another observation that should be further examined is that tumor samples with HBV integrations in the *KMT2B* gene do not have HBV integrations in the *TERT* gene and vice versa [115].

### 8.4. Genes Involved in the Stem Cell Differentiation (GO:0048863)

The genes *SOX5* and the *FN1* are involved in stem cell differentiation while being recurrently affected by HBV DNA integrations. The *SOX5* gene encodes a member of the SRY-related HMG-box (SOX) family of transcription factors, which play important roles in the maintenance of embryonic stem (ES) cell self-renewal and pluripotency. This gene is involved in the regulation of embryonic development and determination of the cell fate, and it has also been suggested that it possibly acts as a transcriptional regulator after the formation of a protein complex.

It has been associated with various types of cancer, including HCC, gastric, breast and prostate cancer. HBV integrations within the *SOX5* have been reported mostly in non-tumor samples. In general, the expression of the *SOX* gene family is frequently down-regulated in gastric cancer and HCC [143], while studies focusing particularly on the *SOX5* gene reported up-regulation in HCC tissues and cell lines as well as in gastric cancer [144,145]. Up-regulation and over-expression of the *SOX5* gene has been associated with metastasis and lower progression-free survival [145].

The *FN1* gene encodes the soluble plasma fibronectin-1 and the insoluble cellular fibronectin-1 proteins. The soluble plasma fibronectin-1 protein is produced by the liver cells, and it is released into the bloodstream where it is involved in blood clotting and wound healing while it also assists in the formation of the extracellular matrix. Moreover, both fibronectin-1 proteins take part in the expansion, migration and differentiation of the cells. *FN1* has been described as a cancer-related gene.

Mutations at the *FN1* gene have been associated with fibronectin glumerulopathy—a kidney disease that results in irreversible kidney failure, while there is an association with various types of cancers, including liver cancer but mostly thyroid cancer. HBV integrations within the *FN1* gene have been reported mostly in non-tumor samples, and it has been found that the gene is downregulated in tumor samples [82].

These observations imply that HBV integrations occur in normal liver tissue but are not directly associated with HCC [82]. Detected HBV-*FN1* fusion transcripts have been associated with the 5′ side of the *FN1* exons [123] and are predicted to cause alternative splicing [146]. Given that *FN1* is a key modulator of fibrosis [147], the HBV-*FN1* fusion protein is possibly involved in the pathogenesis of liver fibrosis [123]. Moreover, HBV integrations in the *FN1* gene occur preferentially in cases with a high liver fibrosis stage rather than in cases with a low fibrosis stage [122].

### 8.5. Genes Involved in the Wnt Signaling Pathway (GO:0016055, GO:0198738)

Three genes recurrently affected by HBV DNA integrations are involved in the Wnt signaling pathway and cell–cell signaling. These genes are *CCNE1*, *TERT* and *PRKN*. Specifically, the *PRKN* gene is involved in negative regulation of the Wnt signaling pathway (GO:0090090), while the *TERT* gene is involved in positive regulation of the Wnt signaling pathway (GO:0030177).

The *PRKN* gene encodes the parkin protein, a member of the E3 ubiquitin ligases group, that plays a role in the cell machinery by degrading damaged and excess proteins after tagging them with ubiquitin molecules. Ubiquitin-tagged unneeded proteins are moved to the proteasome for degradation. Moreover, the ubiquitin–proteasome system regulates the availability of proteins that are involved in several critical cell activities, including the timing of cell division and growth, and thus it was proposed that parkin acts as a tumor suppressor protein [148].

The *PRKN* gene is an extremely large gene (1.5 Mb) that is located within the FRA6E—the third most active common fragile site [149]. It is frequently altered in ovarian, breast and hepatocellular carcinomas, while it has also been associated with Parkinson and Hansen (also known as leprosy) diseases. Moreover, it has been found that the gene is down-regulated in HCC samples compared to non-tumor samples [148].

### 8.6. Genes Involved in Angiogenesis (GO:0001525)

The *FN1*, *TERT* and *GTF2I* genes are involved in angiogenesis or, in other words, the formation of new blood cells, and they have been found to be affected by HBV DNA integrations. The *GTF2I* gene belongs to the general transcription factors family and encodes the TFII-I transcription factor, which has been associated with the coordination of cell growth and division as well as the control of the calcium flow into the cells. TFII-I is ubiquitously expressed in human cells. It is localized at the cytoplasm, and it translocates to the nucleus at the time of transcription [150].

Genomic alterations within the *GTF2I* gene have been recurrently associated with thymic epithelial tumors and sporadically with other types of cancer [151,152,153,154,155]. HBV integrations within the *GTF2I* gene have been reported mostly in non-tumor samples, while there is no evidence regarding the effect of the integrations in the gene expression values.

### 8.7. Genes Involved in Blood Circulation (GO:0008015)

The *CHRM3*, *PDE3A* and *CPS1* genes are involved in blood circulation and are affected by HBV DNA integrations, while the *ALB* and *FN1* genes are directly related to the blood as blood microparticles (GO:0072562).

The *PDE3A* gene encodes a member of the cGMP-inhibited cyclic nucleotide phosphodiesterase (cGI-PDE) family, whose role is the hydrolyzation of cAMP and cGMP and, thus, is a key point in the regulation of intracellular cyclic nucleotide signals [156]. *PDE3A* has been associated with breast cancer [157], lung adenocarcinoma, cervical carcinoma, melanoma [158] and HCC [159], and it has been found to be overexpressed when compared to non-tumor samples [158,159]. HBV integrations are mainly reported in tumor samples.

The *CHRM3* gene encodes a member of the G protein-coupled receptor family, with the ability of acetylcholine binding and, consequently, the inhibition of adenylate cyclase and degeneration of phosphoinositide mediation of potassium channels and, generally, synaptic transmission. *CHRM3* overexpression has been associated with endometrial carcinoma [160], colorectal cancer [161,162], breast cancer [163], prostate cancer [164], lung cancer [165] and gastric cancer [166]. HBV integrations are mostly reported in non-tumor samples, while the role of this gene in HCC as well as the effect of the integrations in gene expression values remains unknown.

The *CPS1* gene encodes the liver-specific rate-limiting carbamoyl phosphate synthetase I enzyme, which participates in the urea cycle and facilitates the efficient removal of ammonia from the body. Specifically, carbamoyl phosphate synthetase I is the enzyme that catalyzes the first step of the urea cycle in a reaction in which excess nitrogen compounds are incorporated into the cycle to be processed.

Furthermore, research found that *CPS1* knockdown reduced the cell growth [119,167] and decreased the metabolite levels associated with nucleic acid biosynthesis pathway [167]. HBV integrations in the *CPS1* gene have been found mostly in non-tumor samples. The expression levels of *CPS1* in HBV-integrated genomes has not been studied, although studies regarding the role of *CPS1* in HCC suggest that, in HCC samples, the *CPS1* is hypermethylated, and the gene is down-regulated [121].

*ALB* encodes the most abundant protein in the blood plasma, the albumin, and, as a consequence, it has an enhanced role in the liver. The encoded protein functions in the regulation of the blood plasma colloid osmotic pressure and acts as a carrier protein for a wide range of endogenous molecules. *ALB* expression has been reported to be restricted in the liver, and mutations at this gene have been associated with liver cancer. HBV integrations that disrupt the *ALB* gene have been reported in multiple studies, and it has been shown that they more commonly occur in non-tumor samples than in tumor samples. As for the expression levels, it has been reported that the gene is up-regulated and highly expressed in tumor samples with HBV integrations [130].

### 8.8. Other Protein-Coding Affected Genes

The *SH3RF3* gene encodes the SH3 domain containing ring finger 3, also known as the POSH2 protein (plenty of SH3 domains protein 2). This particular protein has been recently identified, and it has four Src homology 3 (SH3) domains and a Ring finger domain [168]. *SH3RF3* has been associated with acute lymphoblastic leukemia [169], HIV-associated neurocognitive disorder [170], late-onset familial Alzheimer’s disease [171] as well as breast cancer [172]. Although, the functional role and mechanism of *SH3RF3* in pathological processes, especially in cancer, are largely unexplored [172]. HBV integrations have been reported to affect the *SH3RF3* gene, and HBV integrations are more frequent in tumor-derived samples.

The *KCNT2* gene belongs to a large gene family whose members encode proteins for the generation of potassium channels. The *KCNT2* gene encodes a subunit of a voltage-gated potassium channel named SLICK (sequence like an intermediate calcium channel) that is activated by an increase of cytoplasmic Na+ concentration and contributes to slow after-hyperpolarization in certain neuronal populations in the brain [173]. This gene has been associated with various types of cancer, including skin melanoma. HBV integrations have been reported to interrupt the *KCNT2* gene mostly in non-tumor samples. Regarding HCC, it has been found that the *KCNT2* gene is up-regulated when compared with normal samples [174,175], but there is no other evidence regarding the role of HBV integrations in the observed overexpression of the gene in HCC samples.

The *LINGO2* gene belongs to the *LINGO* gene family and encodes a novel protein that has a role in the central nervous system during early developmental stages as well as in the limbic system and cerebral cortex in adult tissues. The role of the gene in health and disease remains unclear as it has been reported to play a role in multiple diseases including asthma [176], chronic obstructive pulmonary disease [177], Parkinson’s and inflammatory bowel disease [178], as well as various types of cancer (esophageal squamous cell carcinoma, gastric cancer, pancreatic cancer, renal cell carcinoma, lung cancer, breast cancer, bladder cancer and prostate cancer) [179,180,181]. Specifically, in gastric cancer, *LINGO2* up-regulation has been associated with advanced clinical stage and decreased Overall Survival (OS) [180].

### 8.9. Long Non-Coding RNA Genes (lincRNAs)

The *LINC00486* and *FAM157A* genes are long non-coding RNA genes (lincRNAs) that are recurrently affected by HBV integrations. The *LINC00486* gene is a member of the Long Intergenic Non-Protein Coding RNAs (LINC) group, while the *FAM157A* gene is a member of the Long non-coding RNAs with the FAM root symbol. HBV integrations within the *LINC00486* gene are more frequent in non-tumor samples, while HBV integrations within the *FAM157A* have been found almost only in tumor samples.

There is no extended bibliography regarding the role of those two genes in tumor progression and association with HCC. Although, in the last couple of years, there has been a growing body of evidence that lincRNAs are induced by cellular stress, thus, promoting cell proliferation and the invasion of various types of cancer [119,182,183]. Moreover, the functional impact of mutations, genomic alterations and chromosomal deletions, insertions and translocations in these genes is unknown. Although, it has been proposed that genetic alterations at the genomic sites referring to lincRNAs may influence the binding affinity for proteins and possibly result in higher-order structure of the lincRNAs [184].

It should be also mentioned that the interplay between HBV and generally viral infections and the miRNA machinery of the host is a subject of intensive investigations, as there is evidence of existence of viral mechanisms that lead to reprogramming of the miRNA machinery [185]. Specifically, the HBV X protein triggers selective miRNA downregulation [186] while at the same time, HBV integration sites within miRNA genes (*MIR4457* and *MIR4424*) have been reported [115].

Moreover, associations of the number of HBV integration sites with the patient survival have been reported. Specifically, a positive association between the large numbers of integration sites and the increased serum HBsAg and alpha-fetoprotein (AFP) levels has been reported, while, at the same time, cases with HCC and large numbers of HBV integrations have been reported to survive a significantly shorter time than those with no or low numbers of HBV integrations [82]. Finally, individuals with HBV integrations seem to develop HCC at younger ages [82].

Analysis of the correlation between HBV genotypes and their integration frequencies would be necessary to understand whether they exhibit different capacity of integration. At the same time, the role of HBV integration events in different subclasses of HCC should also be investigated. Moreover, different technologies might have different false positive and false negative HBV integration identification rates.

False Positive (FP) results can be obtained from different methodologies used during library preparation and ligation procedures, while the False Negative (FN) rate may be related to the sequencing depth, quality as well as the mapping parameters. Another major issue is the bioinformatics methodology used to declare an integration event and, in brief, the number of supporting mapped reads and whether these reads are concordantly mapped paired-end reads or chimeric reads consisting of both viral and host genomic parts.

## 9. Molecular Mechanisms of Hepatocarcinogenesis Caused by HBV Integration

The oncogenic contribution of HBV DNA integration in HCC is not yet clear. It has been proposed that the insertion of viral DNA into cellular genomic regulatory regions and coding regions results in the modification of gene expression (cis-activation) and production of structurally and functionally aberrant cellular or hybrid proteins. Specifically, HBV can promote HCC in a variety of ways, including the accumulation of genetic damage due to immune-mediated hepatic inflammation [188,189], the induction of oxidative stress [101,190,191,192], a virus-specific mechanism that involves the HBx and HBs viral proteins [193,194,195,196], epigenetic modifications [197,198] and regulation of miRNA expression [186,199,200].

The role of immunity in inflammation, the progression of fibrosis and hepatocarcinogenesis has been also extensively studied. HBV is considered to be a non-cytopathic virus, and the hepatocellular damage observed during HBV infections is mediated by the host’s immune response to the virus [201]. Specifically, HBV induces little or no innate immune responses and is regulated—without being eliminated—by HBV-specific CD4+ and CD8+ T cell responses and neutralizing antibodies [202,203]. Dysregulation of these adaptive immune responses results in chronic liver injury and subsequently HCC. At the same time, there are findings that support that HBV may be directly cytopathic in conditions of severe immune suppression, albeit the underlying mechanisms of the cytopathic effect have not yet been defined [204,205].

Furthermore, recent studies suggest that core promoter mutations at T1762/A1764 of the HBV genome are associated with a higher risk for developing HCC [206], particularly in young and non-cirrhotic individuals infected with HBV genotype B or C [207], as well, in HBV genotype C infections, the development of HCC can be predicted by mutations/deletions in the preS region [208,209]. Another way that HCC can be promoted after HBV infection is through insertional mutagenesis induced by the integration of HBV DNA into the host genome.

Targeted viral genome integration may lead to the activation of cellular genes with oncogenic potential [210], while up-regulation of the target genes expression endows the carrier hepatocytes with a selective growth advantage and clonal expansion leading to malignancy [105,211,212]. Moreover, genetic instability triggered by HBV integration is considered an important contributor in the pathogenesis of HCC [213,214].

Recently, it has been suggested that HBV integrations serve as the relevant source of HBsAg in the context of chronic infection [215], while elevated levels of HBsAg (≥100 IU/mL) are associated with an increased risk of HCC development [216]. Additionally, elevated HBsAg levels lead to HBV-specific B and T cells exhaustion and weakening of their response [217,218] and, as a result, inflammation in the liver microenvironment that further induces HCC development.

Generalized genomic instability, gene and chromosomal deletions or translocations, the amplification of cellular DNA as well as the generation of fusion transcripts are some major genetic alterations that have been associated with HBV integration sites and have been found to be key points in the progression of HCC [14,111]. These alterations presumably lead to the selection of clones of hepatocytes with a growth advantage.

As a consequence, invasive neoplastic clones with multiple alterations arise, and this whole process results in the deregulated cell proliferation and suppressed apoptosis that is mandatory for autonomous cell expansion. This whole process is enhanced by the increased rate of liver cell turnover that occurs during chronic HBV infection leading to cellular senescence, telomere shortening and generalized genomic instability that contributes to the frequency of additional primary integration events as well as secondary genomic rearrangements occurring at the sites of HBV integrations [14].

HBV integration events could also result in a substantial enhancement of oncogenic or anti-apoptotic signals that possibly have a profound influence on the susceptibility of the liver to carcinogenesis [14]. The major cellular process for the development of cancer is unconstrained cell proliferation, which depends on the balance between cell growth activity and apoptotic signaling pathways. Oncogenic molecules with the ability to promote cell proliferation are capable of inducing apoptosis and, consequently, have the homeostatic capability to limit their own proliferative effects.

Moreover, these molecules have the ability to inhibit the pro-apoptotic programs and disrupt the innate homeostatic mechanism, in a process that results in unregulated proliferation [219]. Due to the presence of complex network molecules within both the oncogenic and anti-apoptotic programs, HBV integration sites into different genomic locations likely affect different molecules while having similar results on the overall cellular neoplastic phenotype [14].

Moreover, HBV integrations that occur within or near non-coding repetitive elements, including Long Interspersed Nuclear Elements (LINEs), Short Interspersed Nuclear Elements (SINEs), Alu elements (named after the restriction enzyme from which those sequences are cut) and Long Terminal Repeats (LTRs) of Endogenous Retroviruses (ERVs), have also been associated with the progression of HCC [15,82,107]. Specifically, HBx-LINE1 chimeric transcripts have been identified in HCC cases and have been associated with poor outcome of the individuals that carry them [96]. The *HBx*-LINE1 chimeric transcript acts as a long-non-coding RNA (lncRNA) with significant oncogenic properties additional to the protein production [96].

## 10. Conclusions

HBV, a 3200 bp-length hepatotropic virus of the Hepadnaviridae family is the main ‘culprit’ of viral hepatitis (along with the Hepatitis C Virus—HCV). In terms of mortality, viral hepatitis is on par with AIDS, malaria and tuberculosis or, in other words, among the top four global infectious diseases. In 2016, the Wold Health Organization (WHO) committed to eliminating viral hepatitis as a public health threat by 2030 (apps.who.int/iris/bitstream/handle/10665/206453/WHO-HIV-2016.04-eng.pdf), while on 25 June 2021, during the ongoing SARS-CoV-2 pandemic, guidance on how HBV elimination should be validated (www.who.int/publications/i/item/9789240028395) was first released.

With only 9 years to go until the 2030 deadline is reached, there are still some important key points that should be considered [220]. HBV DNA integration is a major key point regarding the elimination of the virus as well as a burden in the development of a sterilizing cure for HBV as viral proteins are continually expressed even when the HBV DNA is no longer replicating [221]. The cccDNA, the state in which the HBV DNA exists in the nucleus of the infected hepatocytes and serves as a template of HBV DNA replication is minimally inhibited by currently available treatments [220].

Moreover, the high percentage of maternal–fetal (or mother-to-child or vertical) transmission, the minimal screening of HBV infected mothers along with the inability to access medical care facilities and anti-HBV agents for HBV-infected mothers with high HBV DNA levels to prevent vertical transmission especially in developing countries [222,223], the low percentage of HepB3 immunization coverage among one-years-olds that is presented in some regions, as well as the gap in knowledge on whether HBV DNA integrations are inherited in a mendelian fashion—like the Endogenous Retroviruses (ERVs)—or not, further complicate the elimination process.

Additionally, the relationship between HCC and HBV DNA integrations in various host genomic locations is under thorough investigation, while the role of HBV genotypes and sub-genotypes in the integration process along with the role of HBV integration events in different subclasses of HCC should be further studied. Research on the HBV DNA integrations will further enlighten the multistep development of liver cancer and the cooperative mechanisms during neoplastic transformation and will provide evidence for treatment strategies targeting HBV integrations in order to eliminate chronic hepatitis and, thus, decrease the percentage of cirrhosis and HCC.

## Figures and Tables

**Table 1:** The main target human genes affected by >20 reported HBV integrations.

Gene Symbol	Gene Full Name	Gene Type	External Link	Genomic Location	Times Reported	References
*TERT*	Telomerase reverse transciptase	protein coding	HGNC:11730	5p15.33	457	[82,83,87,96,105,107,114,115,116,117,118,119,120,121,122,123,124,125]
*FN1*	Fibronectin 1	protein coding	HGNC:3778	2q35	283	[82,83,84,87,105,107,115,118,119,121,122,123,125,126,127]
*KMT2B*	Lysine methyltransferase 2B	protein coding	HGNC:15840	19q13.12	243	[82,83,84,87,107,115,117,118,119,121,122,123,124,125,126,128,129]
*ALB*	Albumin	protein coding	HGNC:399	4q13.3	70	[83,84,87,107,117,118,119,122,123,125,129]
*CCNA2*	Cyclin A2	protein coding	HGNC:1578	4q27	36	[83,107,118,119,122,123,124,125,130]
*LINC00486*	Long intergenic non-protein coding RNA 486	ncRNA	HGNC:42946	2p22.3	36	[82,83,118,125]
*CPS1*	Carbamoyl-phosphate synthase 1	protein coding	HGNC:2323	2q34	34	[82,83,107,118,119,121,122,123,125,126,129]
*SH3RF3*	SH3 domain containing ring finger 3	protein coding	HGNC:24699	2q13	29	[84,125]
*CCNE1*	Cyclin E1	protein coding	HGNC:1589	19q12	27	[82,83,119,121,124,127]
*GLI2*	GLI family zinc finger 2	protein coding	HGNC:4318	2q14.2	27	[84,115,119,121,126]
*KCNT2*	Potassium sodium-activated channel subfamily T member 2	protein coding	HGNC:18866	1q31.3	26	[118,119,121,122,123,125]
*LINGO2*	Leucine rich repeat and Ig domain containing 2	protein coding	HGNC:21207	9p21.1	25	[83,105,107,123,125]
*PRKN*	Parkin RBR E3 ubiquitin protein ligase	protein coding	HGNC:8607	6q26	24	[81,119,123,125]
*FAM157A*	Family with sequence similarity 157 member A	ncRNA	HGNC:34079	3q29	23	[82,83,87,122,125]
*SOX5*	SRY-box transcription factor 5	protein coding	HGNC:11201	12p12.1	23	[81,83,105,107,119,122,123,124,125]
*GTF2I*	General transcription factor IIi	protein coding	HGNC:4659	7q11.23	22	[84,105,118,122,123,125,127]
*PDE3A*	Phosphodiesterase 3A	protein coding	HGNC:8778	12q12.2	22	[82,84,118,122,125]
*CHRM3*	Cholinergic receptor muscarinic 3	protein coding	HGNC:1952	1q43	21	[83,122,125,129]

## Supplementary Materials

The following are available at https://www.mdpi.com/article/10.3390/microorganisms9081787/s1, Figure S1: Cumulative number of HBV DNA integrations in the human genes.

## Abbreviations

The following abbreviations are used in this manuscript:

HBV	Hepatitis B Virus
CHB	Chronic HBV infection
HCC	Hepatocellular Carcinoma
HBeAg	Hepatitis B e antigen
HBcAg	Hepatitis B core antigen
HBsAg	Hepatitis B surface antigen
anti-HBs	Hepatitis B surface antibody
anti-HBc	Total hepatitis B core antibody
IgM anti-HBc	IgM antibody to hepatitis B core antigen
rcDNA	relaxed circular DNA
dslDNA	double-stranded linear DNA
cccDNA	covalently closed circular DNA
pgRNA	pre-genomic RNA
NHEJ	Non-Homologous End Joining
MMEJ	Microhomology-Mediated End Joining
