# Supplementary material for: Hepatitis B Virus DNA Integration, Chronic Infections and Hepatocellular Carcinoma
Source: Microorganisms. 2021 Aug 23;9(8):1787. doi: 10.3390/microorganisms9081787 (PMC8398950; doi:10.3390/microorganisms9081787)
Supplement: Supplementary file 1 [file microorganisms-09-01787-s001.zip › microorganisms-1339903-supplementary.pdf]

Supplementary Material

Hepatitis B Virus DNA integration, chronic infections and Hepatocellular Carcinoma

Maria Bousali [1], George Papatheodoridis [2], Dimitrios Paraskevis [3] and Timokratis Karamitros [1,4,*]

[1] Bioinformatics and Applied Genomics Unit, Department of Microbiology, Hellenic Pasteur Institute,11521 Athens, Greece; mbousali@gmail.com (M.B.)

[2] Department of Gastroenterology, Medical School of National and Kapodistrian University of Athens, "Laiko" General Hospital of Athens, 11527 Athens, Greece; gepapath@med.uoa.gr

[3] Department of Hygiene Epidemiology and Medical Statistics, School of Medicine, National and Kapodistrian University of Athens, 15772 Athens, Greece; dparask@med.uoa.gr

[4] Laboratory of Medical Microbiology, Department of Microbiology, Hellenic Pasteur Institute,11521 Athens, Greece

*Correspondence: tkaram@pasteur.gr, +30-210-6478871 (T.K.)

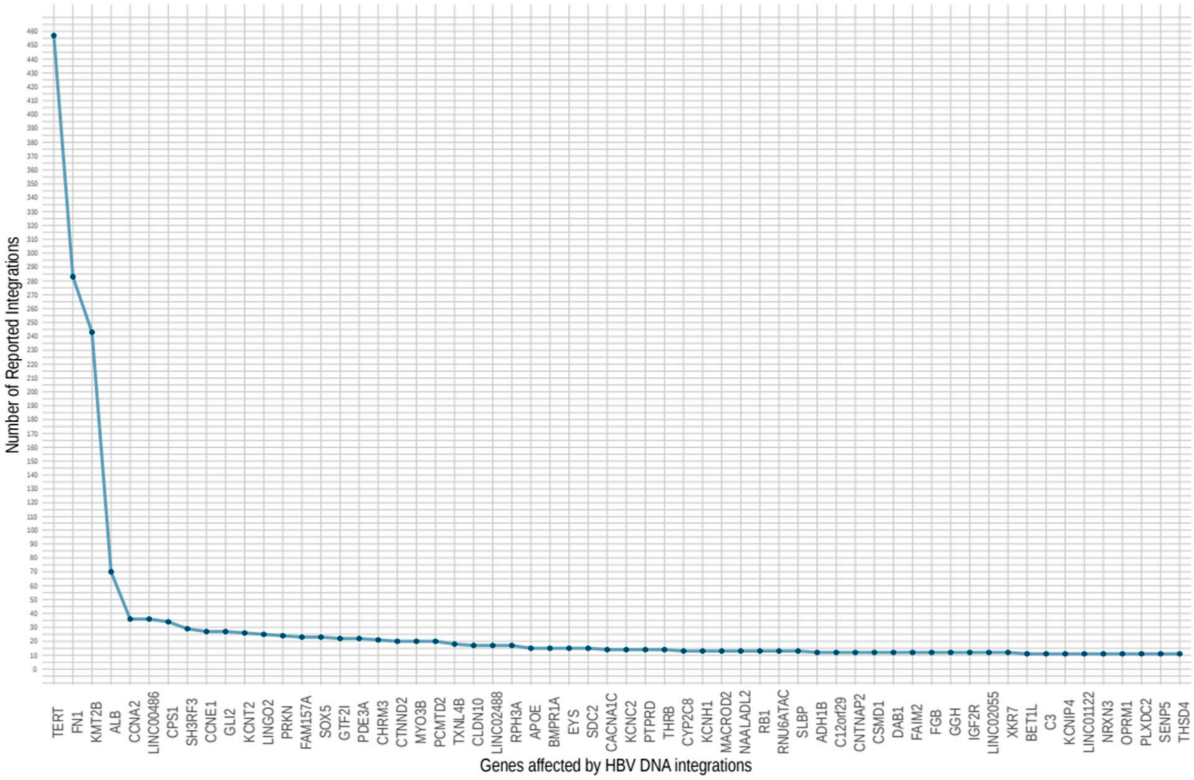

Supplementary Figure S1: Cumulative number of HBV DNA integrations in the human genes (filtered to >10 reported integrations in the literature).
